# Supplementary material for: Model simulations unveil the structure-function-dynamics relationship of the cerebellar cortical microcircuit
Source: Commun Biol. 2022 Nov 14;5:1240. doi: 10.1038/s42003-022-04213-y (PMC9663576; doi:10.1038/s42003-022-04213-y)
Supplement: Supplementary file 2 — Supplementary Information [file 42003_2022_4213_MOESM2_ESM.pdf]

1 Supplementary material

Supplementary figures and videos can be visualized as interactive *.html* files at https://dbbs-lab.github.io/deschepper-etal-2021/, adding e.g. "video" + number + ".html" e.g.: “videoS2.html”, on this URL.

Figure S1 | Placement metrics. Cell placement is assessed using various metrics for each population, including **(a)** Nearest Neighbor distance, **(b)** Pairwise Distance, **(c)** Radial Distribution Function. These metrics show realistic cell positioning.

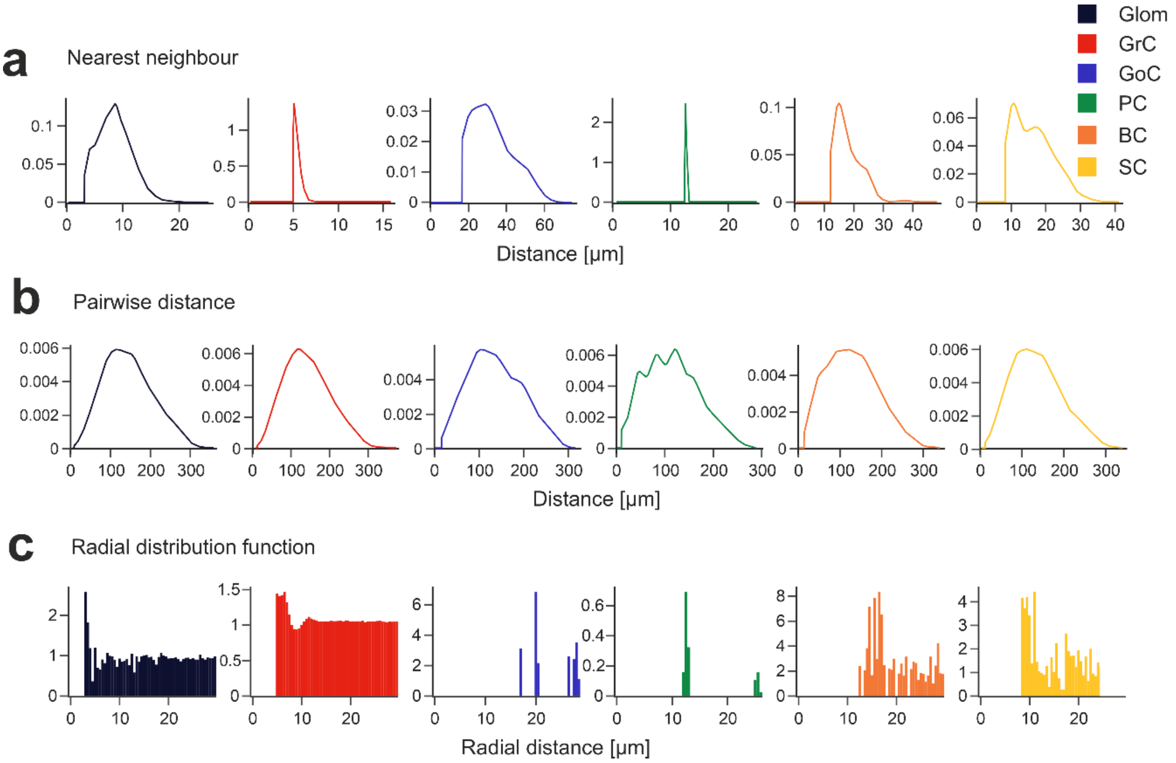

Figure S2 | Connecting *SC-PC* by voxel intersection. A mesh of adjacent voxels is used to enwrap the axon of a stellate cell (50 cubes with 4.6 μm side) and the dendritic tree of a *PC* (50 cubes with 26 μm side). The intersecting voxels are in red. The synapses are located on compartments within the intersecting voxels.

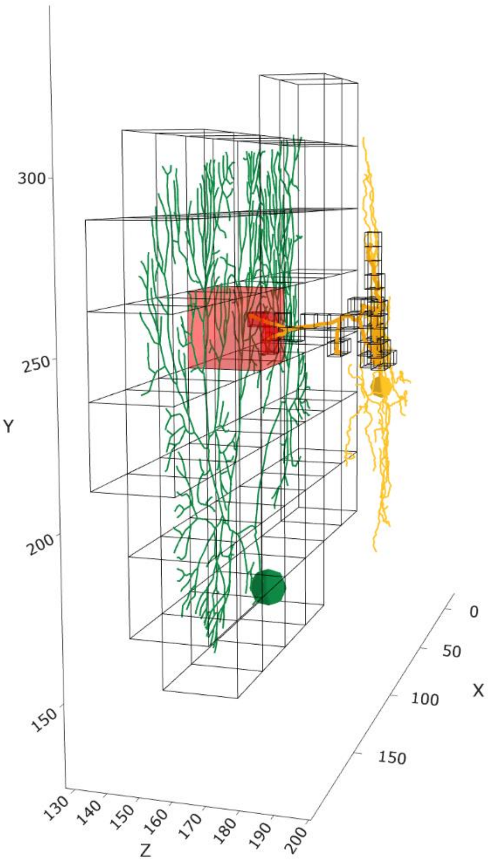

Figure S3 | Coupling graph for *GoCs*. Each of the 70 blue dots represents a *GoC* in the horizontal plane. The grey edges represent connections through gap junctions.

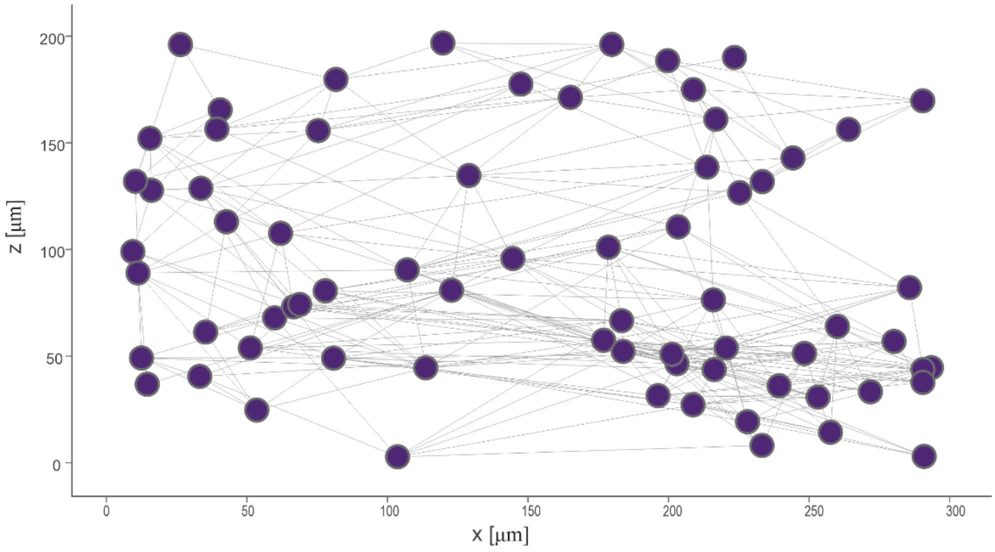

Figure S4 | *MLI* responses to *mf* burst. **(a)** Multiple linear regression of *SC*s and *BC*s in responses to the *mf* burst against the number of synaptic spikes from *pf*s and from other *SC*s or *BC*s. **(b)** One *SC* and one *BC* crossed by an active *pf* beam are represented in 3D. The GABAergic synapses from other *SC*s or *BC*s are also indicated. Bigger markers correspond to presynaptic *GrCs* more activated by the *mf* burst. In this example, the *SC* receives 8% and the *BC* 7.5 % of their *pf* synapses from *GrCs* with at least 2 active dendrites.

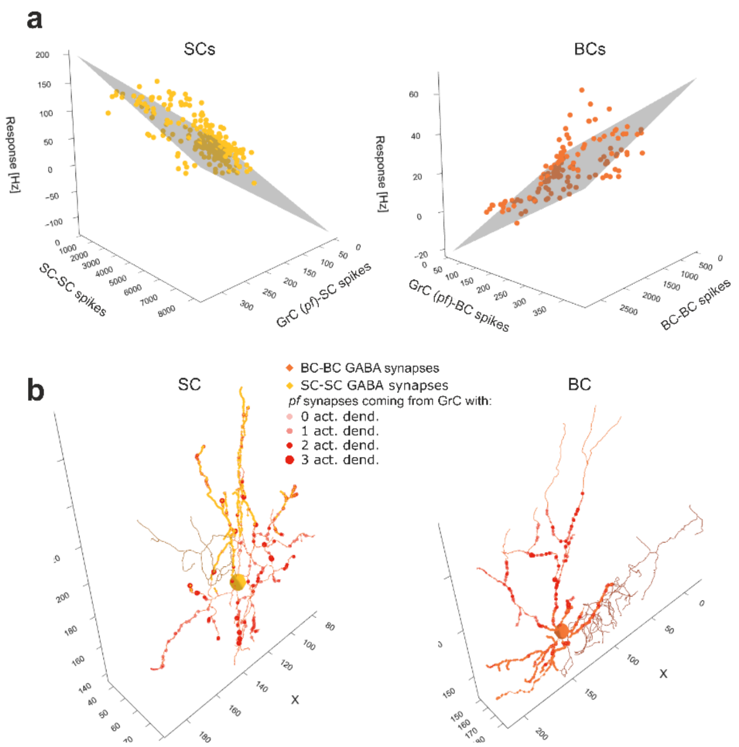

Table S1 | Input-output dependency of model neurons. Multiple linear regression between neuronal responses (firing rates) to the *mf* burst and the number of incoming spikes from the presynaptic neurons (during 40 ms from the *mf* burst onset), averaged over 10 simulations. R^2 and direction coefficients are reported.

	R^2		coefficient
**GrC**	0.91	Glom<br>GoC	6<br>-0.55
**GoC**	0.81	Glom<br>GrC (aa)<br>GrC (pf)<br>GoC	0.14<br>0.15<br>0.1<br>-0.006
**PC**	0.95	GrC (aa)<br>GrC (pf)<br>SC<br>BC	0.057<br>0.091<br>0.074<br>-0.2
**SC**	0.79	GrC (pf)<br>SC	0.54<br>-0.018
**BC**	0.72	GrC (pf)<br>BC	0.11<br>-0.017

Table S2 | Passive properties of the neuron models. Membrane capacitance $\mathbf{C_m}$; Axial resistance $\mathbf{R_a}$ Leakage conductance $\mathbf{G_L}$; Passive conductance for myelinization **Pas**; Reversal potential for leakage $\mathbf{E_L}$. They were based, if the data was available, on experimental data and adapted during the single cell fitting procedure. The Pas channel was used only in *PC*s to simulate the myelin sheath. The values for *GrC*, *GoC* and *SC* are the same published in the respective papers [16,17,19]. The values for the *PC* were adapted to mice morphologies and, as such, are slightly different compared to [109]. The mouse *BC* and *PC* models are not published yet.

The following links contain the passive and active (ionic and synaptic) properties and their distribution for each model:

- https://github.com/dbbs-lab/models/blob/master/dbbs_models/granule_cell_models.py

- https://github.com/dbbs-lab/models/blob/master/dbbs_models/golgi_cell_models.py
- https://github.com/dbbs-lab/models/blob/master/dbbs_models/purkinje_cell_models.py
- https://github.com/dbbs-lab/models/blob/master/dbbs_models/basket_cell_models.py
- https://github.com/dbbs-lab/models/blob/master/dbbs_models/stellate_cell_models.py

	**C_m** [μF/cm^2]	**R_a** [Ω cm]	**G_L** [S/cm^2]	**Pas** [S/cm^2]	**E_L** [mV]
**GrC**	1 - 2.5	100	3.53e-07 – 0.00029	-	-60
**GoC**	1 - 2.5	122	1e-06 - 3e-05	-	-55
**PC**	1 - 6	122	0.0003 - 0.001	5.6e-09	-61
**BC**	1	122	3e-05	-	-60
**SC**	1 – 1.5	110	8e-06 - 3e-05	-	-48
