# Supplementary material for: Model simulations unveil the structure-function-dynamics relationship of the cerebellar cortical microcircuit
Source: Commun Biol. 2022 Nov 14;5:1240. doi: 10.1038/s42003-022-04213-y (PMC9663576; doi:10.1038/s42003-022-04213-y)
Supplement: Supplementary file 3 — Description of Additional Supplementary Files [file 42003_2022_4213_MOESM3_ESM.pdf]

Description of Additional Supplementary Files

Movie S1 | Impulsive response of the cerebellar network. The movie shows the activation of the cerebellar network receiving (at movie time = 12 s, indicated by a green light) the *mf* burst (20ms@200 Hz on a bundle of adjacent 4 *mf*s) to emulate whisker/facial sensory stimulation *in vivo*, superimposed on diffused background noise [21]. Note the sparse activity in *GrC*s that suddenly aggregates into dense clusters activating the overlaying *PC*s and *MLI*s. This pattern gives rise to a vertical column of activity in the cerebellar cortex (see also Fig.6). It should be noted that, compared to [14], the granular layer occasionally activates clusters of *GrC*s under background bombardment due to the *mf-glom* ramifications and clustering that privileges the areas where *glom*s originating from the same *mf*s overlap. Nonetheless, this activity is rarely strong enough to cause a visible activation of the overlaying *PC*s and *MLI*s.

Movie S2 | *GrC* response. The electrical activity of a *GrC* embedded into the cerebellar network (same cell as in Fig. 5a with "4 active dendrites") is animated along with dynamic changes in molecular and synaptic variables. The movie shows 100 ms of simulation, with the *mf* burst (20ms@200 Hz on a bundle of adjacent 4 *mf*s superimposed on diffused background noise) starting at movie time = 5 s (indicated by a green light). Each variable is reported on the *GrC* morphology (4 dendritic compartments, soma, axon hillock, ascending axon, parallel fiber) using a colour scale. **(V_m)** membrane potential**; (Ca)** intracellular calcium concentration; (**AMPA)**, (**NMDA)** and (**GABA)**, synaptic currents recorded in voltage-clamp.

Movie S3 | *GoC* response. The electrical activity of a *GoC* embedded into the cerebellar network (same cell as in Fig. 5c) is animated along with dynamic changes in molecular and synaptic variables. The movie shows 100 ms of simulation, with the *mf* burst (20ms@200 Hz on a bundle of adjacent 4 *mf*s superimposed on diffused background noise) starting at movie time = 0 s (indicated by a green light).Each variable is reported on the *GoC* morphology (using a colour scale) and shown at the bottom with traces (using a moving time window) referring to specific neuron compartments: the soma (teal), an apical dendrite (pink) and a basolateral dendrite (gold). **(V_m)** membrane potential traces in the soma

and apical dendrite. **(Ca)** $[Ca^{2+}]_{in}$ in the basolateral and apical dendrite. **(AMPA)** synaptic current in the basolateral and apical dendrite (synapses from *mf*s, *aa*s, and *pf*s). **(NMDA)** synaptic current in the basolateral dendrite (synapses from *mf*s and *aa*s). **(GABA)** synaptic current in the basolateral dendrite (synapses from *GoC*s).
